# The Effects of Ultra-High Pressure Combined with Egg White Protein on the Gel Physical Properties of Reduced-Salt Shrimp Surimi

**DOI:** 10.3390/foods14122144

**Published:** 2025-06-19

**Authors:** Yefan Wang, Zisheng Zhai, Xinchi Yu, Deyang Li

**Affiliations:** SKL of Marine Food Processing & Safety Control, National Engineering Research Center of Seafood, Collaborative Innovation Center of Seafood Deep Processing, Liaoning Province Key Laboratory for Marine Food Science and Technology, School of Food Science and Technology, Dalian Polytechnic University, Dalian 116034, China

**Keywords:** ultra-high pressure, egg white protein, sodium-reduced shrimp surimi, gel properties, response surface methodology

## Abstract

This study investigated the synergistic effects of ultra-high pressure (UHP) and egg white protein (EWP) on the gel properties of sodium-reduced shrimp surimi. A Box–Behnken design targeting UHP pressure (200–400 MPa), duration (10–20 min), and EWP/myofibrillar protein (MP) ratio (1:9–5:5) was implemented to optimize gel strength, water holding capacity (WHC), and whiteness. Optimal conditions (290 MPa/15 min/EWP:MP = 3:5) yielded the following validated improvements, versus conventional processing: 282.27 g·mm gel strength, 14.90% WHC enhancement, and 16.63% reduced cooking loss. Texture profile analysis demonstrated superior elasticity in composite gels. Magnetic resonance imaging and scanning electron microscopy revealed a denser microstructure with enhanced water-binding capacity, corroborated by the rheological evidence of strengthened viscoelasticity. UHP promotes the partial expansion of MP, exposing hydrophobic groups and sulfhydryl groups, thereby enhancing intermolecular interactions. It also promotes the expansion of EWP, enabling the formation of disulfide bonds between molecules and facilitating the formation of network structures. These findings propose a scalable strategy for developing clean-label salt-reduced aquatic surimi products.

## 1. Introduction

During the processing of shrimp paste products, it is often necessary to add 2% to 3% sodium chloride (NaCl) to dissolve myofibrillar proteins (MP), promote the formation of a three-dimensional gel network structure, and optimize texture properties [1,2]. However, excessive salt intake is closely associated with an increased risk of chronic diseases such as hypertension [3]. The World Health Organization has explicitly proposed a 30% reduction in global salt intake, and modifying food formulations is a crucial strategy to achieve this goal [4]. Therefore, maintaining the gel quality of shrimp paste products while reducing salt content has become a central challenge in the current development of low-salt foods.

To address the quality bottleneck of low-salt shrimp surimi products, the academic community has investigated various salt reduction strategies. Exogenous additives, such as soy protein isolate and chickpea protein, enhance gel properties by modulating protein interactions [5,6]. Egg white protein (EWP) has garnered significant attention due to its exceptional gelation, water retention, and capacity to form a stable network upon heating, which facilitates the unfolding of MP molecules and strengthens hydrophobic interactions and disulfide bond cross-linking [7,8]. Additionally, physical field processing technologies, including microwave, ultrasound, and ultra-high pressure, modify protein conformation through non-thermal effects, thereby improving the gel properties of low-salt systems while minimizing nutrient loss [9,10,11]. Notably, the synergistic effect of physical fields and externally added substances can mitigate the limitations of individual methods. For instance, ultra-high pressure (UHP) promotes the aggregation of EWP, thereby enhancing gel stability through filling and cross-linking effects, thus offering an innovative approach for salt reduction while preserving texture [12]. Food scientists have demonstrated the feasibility of UHP for large-scale industrial processing [13]. In response to the high demand for fresh ready-to-eat seafood and food safety, UHP can strike a balance between safety, quality, processing efficiency, and regulatory compliance from an industrial perspective and is conducive to the production of clean-label products [14]. 

This study investigates the MP of *Litopenaeus vannamei* by employing a combination of UHP technology and EWP addition. The research systematically examines the synergistic regulatory mechanisms of pressure, time, and EWP ratio on gel properties. The Box–Behnken response surface methodology was employed to optimize the process parameters, focusing on gel strength and water holding capacity (WHC) as the primary indicators. This was further supported by texture, rheological, and microstructural analyses to compare the effects of varying salt contents and processing methods on gel characteristics. By elucidating the multi-scale action mechanisms of the UHP-EWP composite treatment, this study aims to provide theoretical foundations and technical support for enhancing the gel properties of low-salt shrimp surimi products, thus promoting clean-label production. This initiative is intended to drive the transformation of the aquatic product processing industry towards a healthier direction.

## 2. Materials and Methods

### 2.1. Materials and Chemicals

*L. vannamei* (mean weight 17.2 ± 1.8 g) were sourced from Qianhe Market (Dalian, China). All analytical-grade chemicals, including MgCl_2_ (purity ≥ 99%), Na_2_HPO_4_ (purity = 99%), NaH_2_PO_4_ (purity = 99%), and ethylenediaminetetraacetic acid (EDTA) (purity ≥ 98%), were obtained from BIOCITY Co., Ltd. (Beijing, China). Frozen shrimps were thawed at an ambient temperature (25 ± 1 °C) and subsequently processed by manually removing the shells and heads. The extracted shrimp tail meat (STM) was kept at −30 °C until experimental use. Egg white protein (EWP) powder (protein content 811 mg/g dry weight) produced by Suzhou Ovopharm Egg Products Co., Ltd. (Suzhou, China) was selected for this investigation.

### 2.2. Extraction of Shrimp MP

The MP was extracted from *L. vannamei* following the modified protocol established by Li et al. [15]. The STM was homogenized in a 1:4 (*w*/*v*) ratio with a phosphate buffer containing 100 mM of NaCl, 1 mM of EDTA, 100 mM of Na_2_HPO_4_/NaH_2_PO_4_, and 2 mM of MgCl_2_, adjusted to a pH of 7.0. A digital homogenizer (Model T25, IKA® Werke GmbH & Co. KG, Staufen, Germany) was operated at 10,000 rpm for 20 s, employing three intermittent cycles. The homogenate was then subjected to sequential filtration through double-layered 80-mesh sterile gauze, followed by centrifugation at 10,000× *g* for 10 min at 4 °C to eliminate soluble fractions. The resultant protein pellet was resuspended in a 0.1 M NaCl solution at four times the pellet volume and rehomogenized under the same conditions. A subsequent centrifugation at 10,000 × g for 10 min at 4 °C produced purified MP precipitates, which were promptly stored at 4 °C and analyzed experimentally within 24 h post-extraction.

### 2.3. Sample Preparation for Single-Factor Experiments

#### 2.3.1. Preparation of Protein in Different Proportions

The MP solutions were proportionally blended with EWP powder at mass ratios of 1:9, 1:4, 3:7, 2:3, and 1:1 (corresponding to EWP mass fractions of 10–50% in total protein content). The composite solutions were adjusted to 50 mg/mL protein concentration using NaCl-supplemented deionized water (1.5% *w*/*w* NaCl). Three homogenization cycles (20 s each at 10,000 rpm) were conducted using a digital homogenizer, with ice bath intervals to prevent thermal denaturation. Homogenized samples were equilibrated at 4 °C for 12 h prior to ultra-high pressure (UHP) processing.

The UHP treatment was performed in a sterilization-grade chamber (Model L2-600/1, Huataisen Miao Bioengineering Technology Co., Ltd., Tianjin, China) at 300 MPa for 20 min using deionized water as the pressure-transmitting fluid. Process parameters were maintained within ± 2 MPa deviation throughout the pressure-holding phase [16].

#### 2.3.2. Preparation of Proteins Under Different Pressures

The extracted MP solution was combined with EWP powder at a 2:3 mass ratio (equivalent to 40% EWP mass fraction in total protein content). The composite mixture was diluted to 50 mg/mL protein concentration using deionized water containing 1.5% (*w*/*w*) NaCl. Three homogenization cycles (20 s each at 10,000 rpm) were performed using a digital homogenizer, followed by overnight equilibration at 4 °C.

The UHP treatment was conducted in a sterilization-grade processing chamber (Model L2-600/1, Huataisen Miao Bioengineering Co., Ltd., Tianjin, China) under the following sequential pressure levels: 100, 200, 300, 400, and 500 MPa (20 min holding time per condition). Deionized water served as the hydrostatic pressure-transmitting medium, with the system temperature maintained at 25 ± 1 °C through integrated thermoregulation [17].

#### 2.3.3. Preparation of Proteins at Different Pressure Times

The MP solution was homogenized with EWP powder at a 2:3 mass ratio (equivalent to 40% EWP mass fraction in total protein content). The composite mixture was adjusted to 50 mg/mL protein concentration using NaCl-supplemented deionized water (1.5% *w*/*w* NaCl). Three intermittent homogenization cycles (20 s each at 10,000 rpm) were executed using a digital homogenizer, followed by 12 h of equilibration at 4 °C.

The UHP processing was conducted in a sterilization-grade chamber (Huataisen Miao Bioengineering Co., Ltd., Tianjin, China) at 300 MPa with incremental dwell times (10, 15, 20, 25, 30 min). Deionized water served as the hydrostatic pressure-transmitting medium, with the chamber temperature stabilized at 25 ± 1 °C via integrated thermal regulation [17].

### 2.4. Preparation of Protein Gel Samples

The composite protein samples prepared in Section 2.3 underwent thermal-induced gelation through a two-stage heating protocol adapted from Ye et al. [18]. Protein solutions were first incubated in a water bath (Model CF-HH-8, Shandong Xinhua Medical Instrument Co., Ltd., Zibo, China) at 40 ± 0.5 °C for 60 min, followed by secondary heating at 90 ± 0.3 °C for 30 min. The thermally treated gels were immediately quenched in an ice-water slurry (0–2 °C) for 30 min to terminate structural reorganization and were then stored at 4 °C until subsequent analysis.

### 2.5. Whiteness

The colorimetric characterization of protein gels was conducted following the methodology outlined by Chen et al. [19]. A calibrated spectrophotometer (UltraScan PRO, HunterLab Associates, Inc., Reston, VA, USA) was utilized to quantify surface chromaticity parameters. The CIELAB color space coordinates—comprising lightness (L*), red-green chromaticity (a*), and yellow-blue chromaticity (b*)—were automatically recorded through three-point averaging across the gel surface. Instrumental validation was performed daily using certified white and black calibration tiles prior to measurements. The experiment was performed in three independent replicates.

Whiteness (W*) = 100 − [(100 − L*)^2^ + a*^2^ + b*^2^]^1/2^

### 2.6. Gel Strength

Gel strength determination was conducted following the protocol established by Yu et al. [20] with some modifications. A texture analyzer (TA.XT.Plus, Stable Micro Systems Ltd., Surrey, UK) equipped with a cylindrical probe (P/5S, 5 mm diameter) was employed under the following standardized parameters: pre-test speed of 1.0 mm/s, test speed of 1.0 mm/s, post-test speed of 1.0 mm/s, with the trigger force threshold set at 2.0 g. The gel strength (g·mm) was calculated as the product of deformation distance (mm) at structural failure and the corresponding maximum resistive force (g), derived from force–deformation curves using Exponent software (v6.1.16, Stable Micro Systems). The experiment was performed in six independent replicates.

### 2.7. Water Holding Capacity (WHC)

The WHC of protein gels was quantified following the methodology of Zhao et al. [21]. Pre-weighed gel samples (1.00 ± 0.05 g) were sandwiched between two layers of quantitative filter paper and secured in pre-calibrated 15 mL polypropylene centrifuge tubes (Falcon® 352096, Corning Inc., Corning, NY, USA). Centrifugation was performed at 10,000× *g* for 10 min (4 °C) using a refrigerated centrifuge (Model CF-16RN, Hitachi Koki Co., Ltd., Tokyo, Japan) with automatic rotor imbalance compensation. All tubes were precisely weighed before and following centrifugation. WHC was calculated using as follows:

WHC (%) = [(m1 − m2)/(m1 − m3)] × 100

where m1 represents the total weight of the sample and the centrifuge tube, m2 represents the mass of the sample and the centrifuge tube after centrifugation, and m3 represents the weight of the centrifuge tube before centrifugation. The experiment was performed in three independent replicates.

### 2.8. Optimization of Gel Parameters via Response Surface Methodology (RSM)

The critical parameters, specifically the EWP incorporation ratio, UHP pressure level, and pressure-holding duration, were selected based on single-factor optimization studies. A three-factor, three-level Box–Behnken design (BBD) combined with RSM was employed to evaluate the combined effects on the response variables, gel strength and WHC. The independent variables were coded as −1 (low), 0 (central), and +1 (high) levels [22]. The experimental matrix with corresponding factor codification is provided in Supplementary Table S1.

### 2.9. Optimized Sample Preparation and Validation Experiment

Response surface analysis revealed theoretical optima at an EWP:MP mass ratio of 187:313 (37.4% EWP in total protein) and 292 MPa UHP treatment for 15 min, where gel strength and WHC simultaneously reached maximum values. Practical considerations prompted parameter adjustment to EWP:MP = 3:5 (37.5% EWP) and 290 MPa UHP (15 min) to accommodate industrial processing tolerances. The following three validation cohorts were established: (1) MP-1.5%: MP gel with 1.5% (*w*/*w*) NaCl; (2) MP-2.5%: Native myofibrillar gel with 2.5% (*w*/*w*) NaCl; (3) U-E-1.5%: Composite gel (EWP:MP = 3:5) treated at 290 MPa UHP (15 min) with 1.5% (*w*/*w*) NaCl. The comparative analysis of gel strength and WHC was conducted across all groups, with triplicate measurements per condition.

The following five distinct experimental cohorts were established to characterize the interaction system between UHP, EWP, and MP: (1) Con-1.5: MP gel with 1.5% (*w*/*w*) NaCl; (2) U-E: Composite gel (EWP:MP = 3:5) subjected to 290 MPa UHP for 15 min, with 1.5% (*w*/*w*) NaCl; (3) UHP: MP gel processed at 290 MPa UHP for 15 min, with 1.5% (*w*/*w*) NaCl; (4) EWP: Composite gel (EWP:MP = 3:5) containing 1.5% (*w*/*w*) NaCl without pressure treatment; (5) Con-2.5: MP gel with 2.5% (*w*/*w*) NaCl, serving as a control for salt concentration. All formulations underwent a standardized two-stage thermal processing protocol in triplicate, maintaining identical homogenization and equilibration parameters, with the exception of the specified UHP and EWP variables. The nomenclature for the cohorts systematically denotes the following: U = UHP treatment, E = EWP incorporation, Con = control, and the numerical suffix indicates the NaCl concentration.

### 2.10. Cooking Loss

Cooking loss (CL) was determined following the methodology of Huang et al. [23]. Pre-weighed gel samples (w1) were individually sealed in heat-resistant polyethylene pouches. Thermal processing was conducted in a precision-controlled water bath (Model CF-HH-8, Shandong Xinhua Medical Instrument Co., Ltd., Zibo, China) maintained at 100 ± 0.5 °C for 15 min. Subsequently, the pouches were immediately immersed in an ice-water slurry for 5 min, followed by a 30 min equilibration at ambient temperature (25 ± 1 °C). Surface moisture was removed using absorbent filter paper prior to obtaining the post-cooking mass (w2). The experiment was performed in three independent replicates. CL was calculated as follows:

CL (%) = (w1 − w2)/w1 × 100

### 2.11. Moisture Distribution

The transverse relaxation (T2) imaging (MRI) of protein gels was analyzed using a low-field nuclear magnetic resonance (LF-NMR) analyzer (MesoQMR23-060H, Niumag Corporation, Shanghai, China), following established protocols [24]. Cylindrical specimens, measuring 10 mm in height and 6 mm in diameter, were machined using stainless steel coring tools to ensure geometric precision. The experiment was performed in three independent replicates.

### 2.12. Texture Profile Analysis (TPA)

The texture characterization of gels was performed following the methodology of Bai et al. [25]. A texture analyzer (TA.XT.Plus, Stable Micro Systems Ltd., Surrey, UK) equipped with a P/50 probe was operated under the following controlled parameters: pre-test/post-test speed of 5.0 mm/s, test speed of 1.0 mm/s, compression strain of 50%, and trigger force threshold of 5.0 g in `auto-trigger mode. Two consecutive compression cycles were executed to derive TPA parameters (cohesiveness, springiness, resilience). The experiment was performed in six independent replicates.

### 2.13. Rheological Properties

The viscoelastic properties of protein gels were investigated using a strain-controlled rheometer (Discovery Hybrid Rheometer DHR-2, TA Instruments, New Castle, DE, USA), following the methodology of Zhang et al. [16]. A parallel plate geometry was employed, with the upper plate having a diameter of 40 mm and the lower Peltier plate measuring 20 mm, under temperature-regulated conditions maintained at 25 ± 0.1 °C. Frequency sweep oscillatory measurements were conducted within the range of 0.1–18 Hz, which was within the linear viscoelastic region, to determine the evolution of the storage modulus (G′) and loss modulus (G”). Steady shear viscosity profiles were obtained through controlled rate flow experiments, covering a shear rate range of 1–200 s^−1^ and utilizing an anti-evaporation solvent trap. The experiment was performed in three independent replicates.

### 2.14. Microstructure

The morphology of protein gels was characterized using a cold field-emission scanning electron microscope (SEM; SU8010, Hitachi, Tokyo, Japan) equipped with cryo-preparation capabilities. Specimens were rapidly cryofixed in liquid nitrogen slush (−180 °C) and subsequently transferred under vacuum to a cryo-preparation chamber. Freeze-etching was conducted at −90 °C for 30 min. Secondary electron images were obtained at an acceleration voltage of 15 kV using a lower detector in high-vacuum mode. Representative micrographs at 1.00 k× magnification captured cross-sectional network architectures under consistent contrast and brightness conditions [26].

### 2.15. Statistical Analysis

Experimental data were analyzed using one-way analysis of variance (ANOVA) with Student–Newman–Keuls testing, which was conducted using SPSS Statistics v19.0 (IBM Inc., Chicago, IL, USA). Significance level analysis was performed at *p* < 0.05. The outputs from the response surface methodology (RSM) were assessed with Design-Expert v13.0 (Stat-Ease Inc., Minneapolis, MN, USA). Graphical representations of the datasets, including error bar plots and 3D response surfaces, were generated using OriginPro 2018 (OriginLab Corp., Northampton, MA, USA).

## 3. Results and Discussion

### 3.1. Single-Factor Test Results

#### 3.1.1. Effect of EWP Ratio on the Gel Properties of Shrimp Surimi

**Figure 1:**
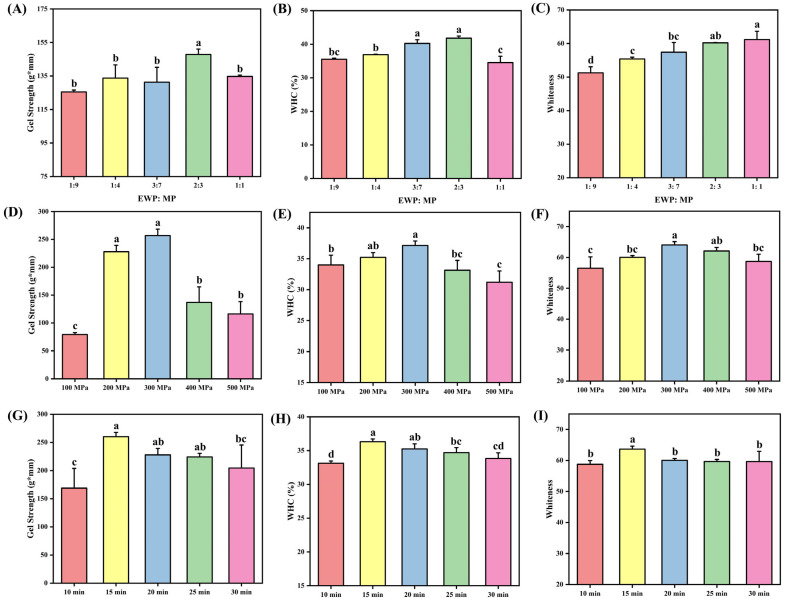
The effect of the ratio of EWP and MP on hybrid protein gel strength (**A**), WHC (**B**), and whiteness (**C**). The effect of UHP pressure on hybrid protein gel strength (**D**), WHC (**E**), and whiteness (**F**). The effect of UHP time on hybrid protein gel strength (**G**), WHC (**H**), and whiteness (**I**). The results are expressed or plotted as mean ± standard deviation. Values of different groups with different lower case letters (a–d) are significantly different at *p* < 0.05.

Figure 1A–C illustrates the gel strength, WHC, and whiteness of composite protein gels subjected to identical UHP conditions while varying the incorporation ratios of EWP. Gel strength, which is a comprehensive indicator of gel texture, serves as a critical parameter for evaluating the mechanical properties of protein matrices [27]. As depicted in Figure 1A, gel strength exhibited a parabolic response to increasing EWP proportions, peaking at an EWP:MP ratio of 2:3. Similarly, WHC, which reflects structural stability through hydration maintenance [28], demonstrated analogous behavior (Figure 1B), achieving maximal retention at the same EWP:MP ratio. The concurrent trends observed in gel strength and WHC suggest a synergistic enhancement of intermolecular crosslinking within the composite gel network due to EWP supplementation [29]. The optimal addition of EWP, at a 2:3 ratio, promoted the formation of a denser network, thereby improving the architecture of water channels and enhancing hydration mediated by hydrophobic interactions. However, excessive EWP (greater than a 2:3 ratio) resulted in protein hyperaggregation during UHP treatment, which disrupted the exposure of hydrophobic domains and compromised gelation efficiency due to steric hindrance and phase separation [30]. Whiteness, a critical quality parameter for gel-based products, exhibited a monotonic enhancement with increasing proportions of EWP (Figure 1C). This enhancement can be attributed to the light-scattering effects resulting from a homogeneous dispersion of proteins. These findings are consistent with those reported by Huang et al. [8], who noted that the modulation of hydrophobic interactions and disulfide bonding in myofibrillar gel systems is dependent on EWP concentration. They observed that excessive EWP leads to the formation of macroaggregates and structural heterogeneity.

#### 3.1.2. Effect of UHP Pressure on the Gel Properties of Shrimp Surimi

Figure 1G–I illustrates the gel strength, WHC, and whiteness of composite protein gels subjected to varying UHP treatments. All parameters exhibited parabolic dependencies on UHP intensity, peaking at 300 MPa. Under moderate pressure, UHP facilitates the denaturation and depolymerization of proteins, which enhances their solubility and allows more free water to interact with proteins, forming bound water. This interaction contributes to a more compact gel network structure during the gelation process [31]. Furthermore, the water molecules surrounding the amino acid residues of egg white protein rearrange, resulting in increased luster and improved gel properties [32]. Conversely, excessive pressure leads to over-denaturation through the cleavage of intramolecular bonds, which disrupts crosslinking efficiency and generates discontinuous matrix architectures. This pressure-dependent behavior is consistent with the findings of Li et al. [33] in chicken myofibrillar composite gels, where UHP treatment initially enhanced WHC, textural properties, and thermal stability through controlled protein unfolding, followed by progressive deterioration at supra-optimal pressures due to irreversible aggregation.

#### 3.1.3. Effect of UHP Time on the Gel Properties of Shrimp Surimi

Figure 1D–F illustrates the gel strength, WHC, and whiteness of mixed protein gels subjected to varying durations of UHP treatment. As shown in these figures, the gel strength, WHC, and whiteness of the mixed protein gels initially increase with longer UHP treatment times, peaking at 15 min before subsequently decreasing. These findings suggest that extended pressurization leads to the rupture of chemical bonds within protein molecules. Additionally, the hydrolytic activity of cathepsins results in the significant hydrolysis of proteins in the surimi, which diminishes the cross-linking effects of myosin heavy chains relative to the degradation effects of MP [34]. Liu et al. [31] indicated that an optimal UHP treatment duration can enhance the gel properties of surimi MP.

### 3.2. Optimization with RSM

Based on the results of the single-factor experiments, gel strength and WHC were selected as response variables. The three maximum values from each group of single-factor experiments were chosen for response surface analysis. Specific parameters are detailed in Table 1.

#### 3.2.1. Effect of EWP-UHP Treatment on the Gel Strength of Shrimp Surimi

**Figure 2:**
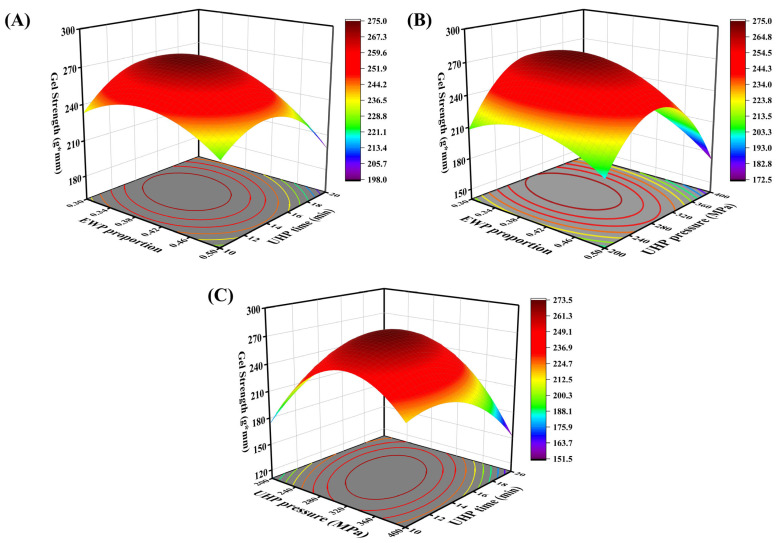
Response surface and contour plots of the interaction of EWP addition (**A**), pressure level (**B**), and holding time (**C**) on gel strength of mixed gels.

The experimental design and results of the response surface are presented in Table 2. Regression fitting was performed on the data in Table 2 using Design-Expert 13 data analysis software. The quadratic multiple regression equation for gel strength (R1) in relation to the three factors EWP ratio (A), UHP pressure (B), and UHP time (C) is as follows: R1 = 273 − 12.04A − 5.39B − 5.34C − 7.72AB − 8.25AC − 26.03BC − 19.98A^2^ − 55.03B^2^ − 29.34C^2^. The significance test and variance analysis of gel strength are presented in Table 3. The results indicate that the experimental model is highly significant (*p* < 0.01). The coefficient of determination (R^2^ = 0.9885) for the regression model suggests a good fit for this quadratic polynomial. Additionally, the coefficient of variation (C.V. = 2.85%) is less than 10%, demonstrating the experimental stability of the regression model. Table 3 shows that the first-order terms A, B, C; the second-order terms AB, AC, BC; and the quadratic terms A^2^, B^2^, C^2^ all significantly affect the gel strength of the mixed protein gel (*p* < 0.05). The factors influencing gel strength, in order of significance, are EWP ratio > UHP pressure > UHP time. Figure 2A–C illustrates the effects of pairwise interactions between EWP ratio and UHP time, EWP ratio and UHP pressure, and UHP pressure and UHP time on the gel strength of low-salt mixed protein gels. As depicted, the pairwise interactions among EWP ratio, UHP pressure, and UHP time all impact gel strength. When other factors are held constant, and within a certain range, the gel strength of the protein gels varies with increases in EWP ratio, UHP pressure, and time. Notably, the curvature of the surface for the UHP pressure–UHP time interaction is relatively larger, indicating a greater interdependence between these two UHP conditions, whereas the influence of the EWP ratio on gel strength appears to be more independent.

#### 3.2.2. Effect of EWP-UHP Treatment on the WHC of Shrimp Surimi

**Figure 3:**
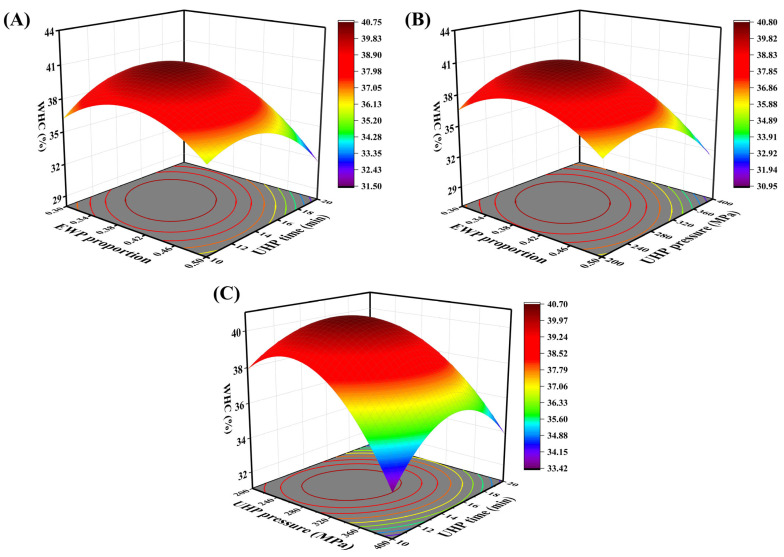
Response surface and contour plots of the interaction of EWP addition (**A**), pressure level (**B**), and holding time (**C**) on the WHC of mixed gels.

The quadratic multiple regression equation for the WHC, denoted as R^2^, is formulated based on the three factors the ratio of EWP (A), UHP pressure (B), and UHP time (C) as follows: R2 = 40.47 − 1.53A − 1.25B − 0.7868C –1.05AB − 1.24AC + 0.9873BC − 2.72A^2^ − 2.95B^2^ − 2.64C^2^. The significance test and variance analysis of WHC are illustrated in Table 4, demonstrating that the experimental model is highly significant (*p* < 0.01). The coefficient of determination (R^2^ = 0.9673) indicates that the quadratic polynomial regression is effective. Additionally, the coefficient of variation (C.V. = 2.40%) is less than 10%, confirming the experimental stability of the regression model. As depicted in Table 4, the first-order terms A, B, and C; the second-order terms AB and AC; and the quadratic terms A^2^, B^2^, and C^2^ all exert significant effects on the WHC of the mixed protein gel (*p* < 0.05). The factors influencing the significance of WHC are ranked in the following order: EWP ratio > UHP pressure > UHP time. Figure 3A–C illustrates the effects of the pairwise interactions between EWP ratio and UHP time, EWP ratio and UHP pressure, and UHP pressure and UHP time on the WHC of low-salt mixed protein gels. The figures indicate that the pairwise interactions among EWP ratio, UHP pressure, and UHP time significantly influence WHC. When other factors are held constant, within a certain range, the WHC of the protein gel exhibits varying changes with increases in EWP ratio, UHP pressure, and time. The curvature of the surfaces representing the interactions between EWP ratio and the other two factors is relatively pronounced, suggesting that the EWP addition ratio has a stronger correlation with other factors regarding WHC and holds a more critical position in the overall system.

#### 3.2.3. Determination and Validation of Optimal Processes

Through the Optimization function in the Design-Expert 13 data analysis software, the optimal process conditions for the UHP-EWP-MP gel were determined as follows: EWP addition constituted 37.4% of the total protein content, with a UHP pressure of 292 MPa and a UHP duration of 15 min, yielding a theoretical maximum gel strength of 274.69 g·mm and a maximum WHC of 40.76%. To enhance practical operation and experimental feasibility, the conditions were adjusted to an EWP:MP ratio of 3:5 (where EWP addition accounts for 37.5% of the total protein content), a UHP pressure of 290 MPa, and a UHP duration of 15 min. Three sets of validation experiments were conducted based on these adjusted conditions, with the experimental results presented in Table S1. The results indicated that the gel strength, WHC, and whiteness of the optimized experimental group were significantly higher than those of both the low-salt and (1.5% salt) normal salt (2.5%) groups.

### 3.3. Effect of EWP-UHP on the Properties of Low-Salt Composite Protein Gels

#### 3.3.1. Gel Strength

**Figure 4:**
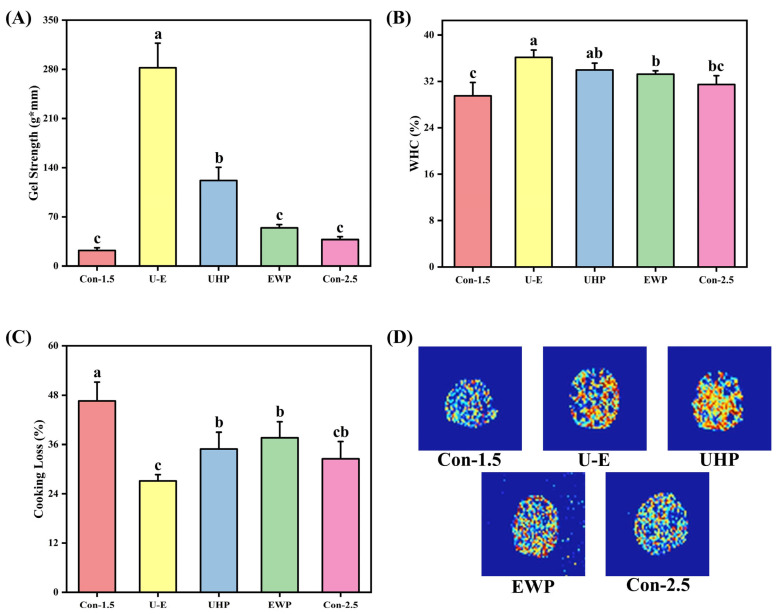
The effects of different treatments on gel strength (**A**), WHC (**B**), CL (**C**), and water imaging (**D**) of mixed gels. Con-1.5, Con-2.5, U-E, UHP, and EWP represent the low-salt group with 1.5% NaCl addition, the high-salt group with 2.5% NaCl addition, the sample group treated with optimized UHP in combination with EWP conditions, and the gel sample group treated with UHP and EWP alone, respectively. The results in Figure 4A–C are expressed or plotted as mean ± standard deviation. Values of different groups with different lower case letters (a–c) are significantly different at *p* < 0.05.

The impact of UHP and EWP on the gel strength of low-salt composite protein is illustrated in Figure 4A. The untreated low-salt group exhibited lower gel strength values compared to the normal salt group, indicating that NaCl can promote the dissolution of myosin and actin. This process reduces electrostatic repulsion between protein molecules, facilitates the unfolding and cross-linking of myosin molecules, and thereby forms a denser gel structure. As noted in the study by Li et al. [35], NaCl enhances ionic strength and shields charges, which improves the solubility and functional properties of myofibrillar proteins, ultimately contributing to a favorable gel structure during heating. The gel strength of both the UHP group and the EWP group was found to be higher than that of the normal salt group, demonstrating that both treatment methods effectively enhance the texture of the gel. UHP promotes the partial unfolding of MP, which exposes hydrophobic and sulfhydryl groups, thereby enhancing intermolecular interactions and forming a more compact three-dimensional network structure. When EWP and MP are heated together, they form a composite gel structure through physical entanglement and chemical cross-linking, filling the voids in the protein gel and enhancing its gel properties [7]. With appropriate UHP and the addition of EWP, the gel strength of the mixed protein gel is significantly higher than that of the other groups, which is consistent with the RSM optimization results. The UHP treatment causes MP to unfold, exposing more binding sites, while also promoting the unfolding of EWP, making it easier for the two to undergo intermolecular cross-linking to form disulfide bonds, thereby synergistically enhancing the gel strength [29].

#### 3.3.2. WHC

The effects of UHP and EWP on the WHC of low-salt composite protein gels are illustrated in Figure 4B. The WHC of the untreated low-salt group is the lowest of all groups, which indicates that the addition of NaCl affects the WHC of egg white gel by increasing the fluidity and distribution of free water [36]. Research conducted by Zhu et al. [1] demonstrated that NaCl facilitates hydration and increases the WHC in meat products. Generally, for protein gels, the trend of WHC correlates with gel strength; both the UHP and EWP groups displayed higher values than the untreated group, consistent with the trend observed in Figure 4A. UHP can influence the molecular structure of certain proteins within muscles, inducing protein depolymerization and enabling more free water to bind with proteins, subsequently altering the WHC of these protein molecules [17]. EWP inherently possesses strong WHC and is commonly utilized as a water-retaining agent in meat product processing. When combined with meat protein, it effectively enhances the gel’s WHC. The U-E group achieved the maximum WHC, which aligns with the results of RSM optimization. The dual-protein network structure treated by UHP plays a reinforcing role in the gel system, further enhancing its WHC.

#### 3.3.3. CL

Figure 4C illustrates the impact of UHP-EWP on the CL of low-salt composite protein gels, where lower values indicate a stronger WHC after cooking. It is evident that the CL of the Con-1.5 group is higher than that of the other groups, demonstrating that the addition of NaCl, UHP treatment, and the incorporation of EWP all enhance the water-trapping ability of MP gels to varying degrees. Among them, the CL in the normal salt group was reduced more significantly, indicating that NaCl promoted protein dissolution and inhibited excessive protein aggregation, thereby reducing the damage caused by local stress due to heating. This improved the binding ability to water, reflecting the important role of NaCl in shrimp paste products [37]. UHP enhances protein cross-linking and water retention properties through physical modification, while EWP reduces the release of free water during cooking by physically filling the moisture channels in the network structure. The U-E group value reached the lowest, indicating that EWP may play a role in maintaining the protein structure during high pressure and heat treatment, preventing excessive water loss caused by oxidative crosslinking [38]. The synergistic effect of EWP and UHP significantly improved the WHC and compactness of the composite gel structure, resulting in reduced CL. According to a report by Niu et al. [39], the mixed gel of soy protein isolate and MP formed a more robust network structure, effectively reducing CL, which is consistent with the findings of this study.

#### 3.3.4. Magnetic Resonance Imaging

The nuclear magnetic imaging of low-salt composite protein gels subjected to UHP-EWP treatment is illustrated in Figure 4D. MRI effectively depicts the distribution of water present in food products. Generally, areas with a higher concentration of hydrogen protons yield brighter proton density maps and redder pseudo-color maps [40]. It is evident that the Con-1.5 group exhibits the largest proportion of blue areas, indicating a looser gel structure. Conversely, the U-E group displays the highest proportion of red areas, suggesting that the water within the composite gel is more uniformly bound, resulting in an overall increase in signal intensity. From a microstructural perspective, UHP allows MP to expand, exposing more hydrophilic groups and synergistically working with the polar groups of EWP to form a denser hydration layer, thereby increasing proton density [7,41]. Additionally, the incorporation of EWP has improved the hydrophilicity of the composite gel, thereby increasing the efficiency of bonding with water molecules. The research conducted by Luo et al. [42] further supports this perspective, as their study on composite surimi gel systems indicated a higher proportion of bound or immobilized water compared to traditional surimi gels, resulting in a more uniform water distribution and a corresponding increase in signal intensity within the system.

#### 3.3.5. TPA

**Figure 5:**
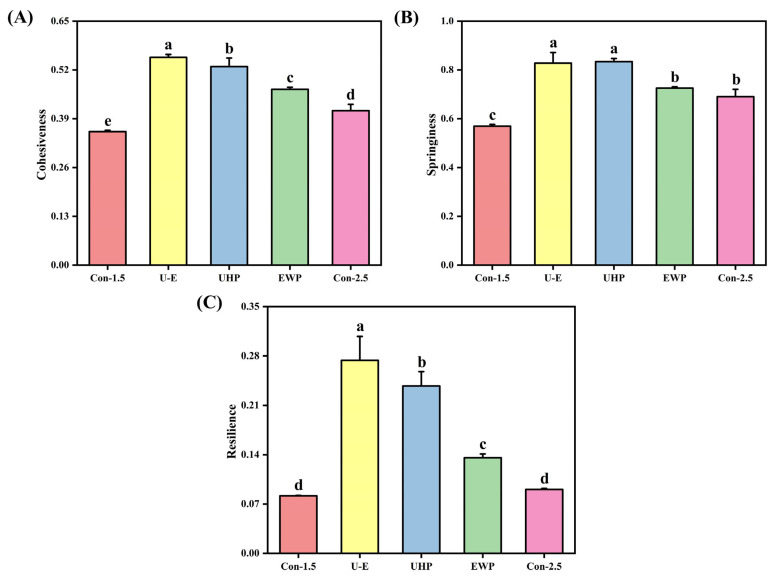
The effects of different treatments on the cohesiveness (**A**), springiness (**B**), and resilience (**C**) of mixed gels. Con-1.5, Con-2.5, U-E, UHP, and EWP represent the low-salt group with 1.5% NaCl addition, the high-salt group with 2.5% NaCl addition, the sample group treated with optimized UHP in combination with EWP conditions, and the gel sample group treated with UHP and EWP alone, respectively. The results are expressed or plotted as mean ± standard deviation. Values of different groups with different lower case letters (a–e) are significantly different at *p* < 0.05.

The effect of UHP-EWP on the texture of low-salt composite protein gels is illustrated in Figure 5, where Figure 5A–C displays the variations in cohesiveness, springiness, and resilience of the gels, respectively. Texture serves as a critical indicator for assessing gel quality, reflecting the interactions between protein–protein and protein–water molecules within the gel [43]. From Figure 5, it is generally observed that the cohesiveness, springiness, and resilience of the gels exhibit similar trends. Overall, the Con-1.5 group exhibited the minimum value, slightly lower than that of Con-2.5, indicating that NaCl can enhance ionic strength, neutralize the negative charges on the MP surface, reduce intermolecular electrostatic repulsion, promote the exposure of hydrophobic groups, and provide cross-linking sites [1]. In contrast, the UHP group exhibited superior textural properties, suggesting a tight binding of proteins facilitated by UHP. The increased ratio of bound water diminishes the fluidity of the gel under external forces, consequently enhancing its cohesiveness. Furthermore, a more uniform network effectively dissipates external force energy through the stretching of molecular chains and the reversible breakage of cross-linking points, thereby demonstrating improved elasticity. The stable distribution of water allows the gel to revert to its original state more rapidly following compression or stretching, indicating superior recovery. The U-E group achieved the maximum value, highlighting the synergistic effect of UHP-EWP. Jiang et al. [44] demonstrated that the incorporation of soybean tissue protein can significantly enhance the textural properties of MP gel, with trends consistent with gel strength.

#### 3.3.6. Rheological Properties

**Figure 6:**
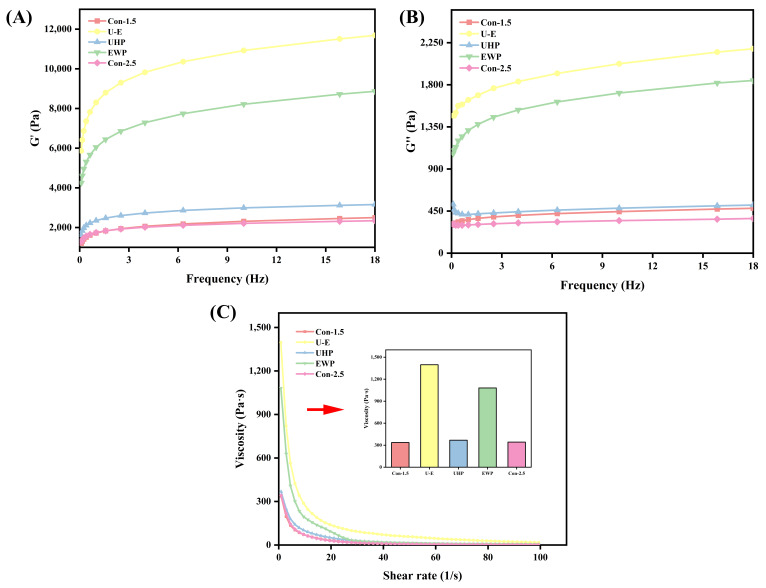
The effects of different treatments on the G′ (**A**), G″ (**B**), and viscosity (**C**) of mixed gels. Con-1.5, Con-2.5, U-E, UHP, and EWP represent the low-salt group with 1.5% NaCl addition, the high-salt group with 2.5% NaCl addition, the sample group treated with optimized UHP in combination with EWP conditions, and the gel sample group treated with UHP and EWP alone, respectively.

The frequency sweep illustrates the gel’s response to varying frequencies and is closely related to its texture. It serves as a means to characterize the type of gel, which can be classified as either a weak gel or a strong gel [45]. As depicted in Figure 6A,B, the overall curves for the U-E group, UHP group, and EWP group are consistently above those of the Con-1.5 group and Con-2.5 group, indicating superior rheological properties; notably, the U-E group achieved the maximum value, aligning with previous experimental findings. All groups demonstrated higher storage modulus (G′) values compared to loss modulus (G″) values under the same frequency sweep, suggesting that the elastic properties of all composite gel samples surpass their viscous properties, thereby confirming the gel characteristics of the samples. The treatment of UHP-EWP enables proteins to form more complex composite networks, and the diversity of cross-linking enhances the network strength, resulting in an increase in G′ [46]. According to the research conducted by Fan et al. [17], both the G′ and G″ of MP gels treated with UHP exhibited significant increases during frequency scanning.

As illustrated in Figure 6C, the overall viscosity curves for the U-E group, UHP group, and EWP group are consistently higher than those of the Con-1.5 group and Con-2.5 group. This trend is more intuitively and clearly represented by the initial viscosity values. The viscosity value of the EWP group was notably high, likely due to the inherently high viscosity of EWP [12]. The U-E group exhibited the highest viscosity value, indicating a greater overall cross-linking density within the processed composite gel. In contrast, the pure MP gel experienced significant thinning at high shear rates due to its fragile network structure, while the composite gel’s network was more resilient, resulting in higher viscosity. Additionally, the study conducted by Gao et al. [47] supports the finding that the composite gel increases the viscosity of the low-salt MP gel as a consequence of alterations in protein conformation.

#### 3.3.7. Microstructure

**Figure 7:**
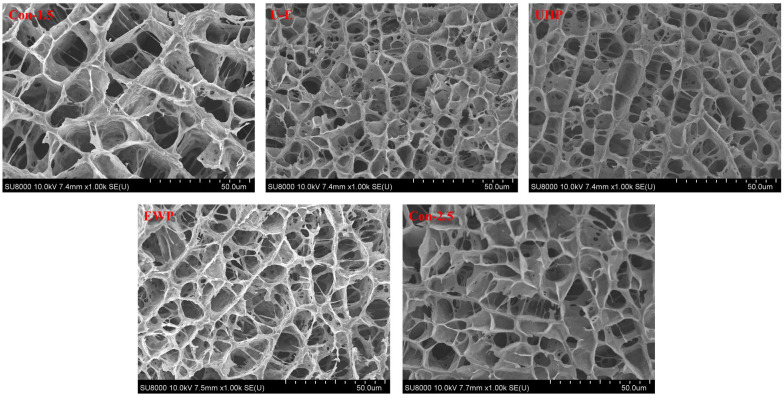
The effects of different treatments on the microstructures of mixed gels. Con-1.5, Con-2.5, U-E, UHP, and EWP represent the low-salt group with 1.5% NaCl addition, the high-salt group with 2.5% NaCl addition, the sample group treated with optimized UHP in combination with EWP conditions, and the gel sample group treated with UHP and EWP alone, respectively.

Figure 7 illustrates the SEM images of low-salt composite protein gels treated with UHP and EWP, with the objective of examining the microstructure of these composite gels. During the heat treatment process, interactions among surimi proteins facilitate the formation of a porous protein network, which significantly affects various critical quality parameters of the resulting surimi products [48]. As depicted in the figure, all sample groups exhibited a three-dimensional network structure. The gel in the Con-1.5 group displayed larger and unevenly distributed network pores. Furthermore, the size of these network pores serves as water channels, indicating the poor WHC of the low-salt MP gel. In contrast to the low-salt group, the structure of the Con-2.5 group appeared more organized, featuring smaller pores that demonstrate the NaCl-promoted protein cross-linking, resulting in a denser gel. The pore size in the UHP group was smaller than that of the other groups not subjected to UHP processing, suggesting that UHP enhances intermolecular interactions among MP molecules, thereby constructing a more regular and denser network. The channel walls of the three-dimensional structure in the EWP group appeared thicker, which reflects the better water distribution and water-holding performance of the EWP group. In contrast, the pores in the U-E group were smaller, characterized by a denser overall network structure and significantly fewer fractures compared to the EWP group. This demonstrates the synergistic cross-linking promotion effect between UHP and EWP, resulting in a more continuous and denser structure with enhanced water binding capacity. Such characteristics improve resistance to potential deformations during preparation, thereby exhibiting greater mechanical stability. Zhao et al. [5] noted in their research that the addition of plant proteins leads to a denser microstructure in surimi gels. Furthermore, the study by Zhang et al. [16] indicated that the MP gel of UHP-treated *L. vannamei* displayed superior WHC, featuring smaller water channels in its microstructure and a more compact overall structure.

## 4. Conclusions

This study employed a Box–Behnken response surface methodology to develop and optimize the salt-reduced shrimp surimi, utilizing three independent variables, which were UHP pressure, UHP duration, and EWP incorporation ratio. The optimized protocol, which involved 290 MPa of UHP pressure for 15 min with an EWP:MP ratio of 3:5, achieved superior gel strength and WHC compared to conventional high-salt formulations, while simultaneously reducing NaCl content by 40%. The characterization of the composite gel demonstrated that moderate UHP treatment induced protein denaturation, facilitating EWP-MP crosslinking. This process promoted protein aggregation, thereby enhancing the gel-forming capacity of MP. Controlled EWP incorporation synergistically improved gel performance through the dual mechanisms of physical void-filling and chemical crosslinking induced by UHP. The resultant composite gel exhibited enhanced water retention, improved rheological properties, and a homogeneous microstructure. These findings advance the understanding of protein–protein interactions under physical field treatments and provide a scalable solution for developing healthier aquatic surimi products with reduced sodium content. Future work will systematically evaluate sensory attributes such as odor, saltiness, texture softness, and overall acceptability. Following this evaluation, the incorporation levels of EWP and the parameters for UHP processing will be optimized based on sensory feedback, with the goal of achieving a balance between functional properties and consumer preferences.

## Figures and Tables

**Table 1:** Factors and levels of Box–Behnken.

Factors	Code Levels		
	−1	0	1
A-EWP proportion	0.3	0.4	0.5
B-UHP pressure (MPa)	200	300	400
C-UHP time (min)	10	15	20

**Table 2:** Design and results of response surface experiment.

	Factor 1	Factor 2	Factor 3	Response 1	Response 2
Std	A:EWP Proportion	B:UHP Pressure (MPa)	C:UHP Time (min)	R1 Gel Strength (g·mm)	R2 WHC (%)
1	0.3	200	15	209.607	37.1757
2	0.5	200	15	197.687	35.7877
3	0.3	400	15	213.728	35.9106
4	0.5	400	15	170.934	30.3080
5	0.3	300	10	235.888	36.0274
6	0.5	300	10	231.573	35.8621
7	0.3	300	20	232.29	36.8198
8	0.5	300	20	194.968	31.7107
9	0.4	200	10	168.356	37.3983
10	0.4	400	10	210.167	33.8075
11	0.4	200	20	219.161	33.9561
12	0.4	400	20	156.849	34.3145
13	0.4	300	15	278.638	38.8762
14	0.4	300	15	271.463	40.5229
15	0.4	300	15	276.742	41.0508
16	0.4	300	15	270.841	41.1916
17	0.4	300	15	267.323	40.6998

**Table 3:** Significance and analysis of variance (ANOVA) of gel strength.

Source	df	Sum of Squares	Mean Square	F-Value	*p*-Value	
Model	9	24,557.49	2728.61	67.12	<0.0001	Significant
A-EWP proportion	1	1160.43	1160.43	28.55	0.0011	
B-UHP pressure	1	232.55	232.55	5.72	0.0480	
C-UHP time	1	228.06	228.06	5.61	0.0497	
AB	1	238.29	238.29	5.86	0.0460	
AC	1	272.37	272.37	6.70	0.0360	
BC	1	2710.38	2710.38	66.68	<0.0001	
A^2^	1	1681.33	1681.33	41.36	0.0004	
B^2^	1	12,750.49	12,750.49	313.66	<0.0001	
C^2^	1	3624.23	3624.23	89.16	<0.0001	
Residual	7	284.55	40.65			
Lack of Fit	3	199.52	66.51	3.13	0.1497	Not significant
Pure Error	4	85.03	21.26			
Cor Total	16	24,842.04				
R^2^	0.9885					
C.V.	2.85%					

**Table 4:** Significance and analysis of variance (ANOVA) of WHC.

Source	df	Sum of Squares	Mean Square	F-Value	*p*-Value	
Model	9	159.38	17.71	23.01	0.0002	Significant
A-EWP proportion	1	18.80	18.80	24.44	0.0017	
B-UHP pressure	1	12.44	12.44	16.17	0.0051	
C-UHP time	1	4.95	4.95	6.44	0.0388	
AB	1	4.44	4.44	5.77	0.0473	
AC	1	6.11	6.11	7.94	0.0259	
BC	1	3.90	3.90	5.07	0.0591	
A^2^	1	31.12	31.12	40.44	0.0004	
B^2^	1	36.75	36.75	47.76	0.0002	
C^2^	1	29.45	29.45	38.28	0.0005	
Residual	7	5.39	0.7695			
Lack of Fit	3	1.93	0.6442	0.7461	0.5784	Not significant
Pure Error	4	3.45	0.8635			
Cor Total	16	164.77				
R^2^	0.9673					
C.V.	2.40%					

## Data Availability

The original contributions presented in the study are included in the article/Supplementary Materials. Further inquiries can be directed to the corresponding author.

## Supplementary Materials

The following supporting information can be downloaded at: https://www.mdpi.com/article/10.3390/foods14122144/s1, Table S1: The results of test and verify.

## Abbreviations

The following abbreviations are used in this manuscript:

UHP	Ultra-high pressure
EWP	Egg white protein
MP	Myofibrillar protein
WHC	Water holding capacity
RSM	Response surface methodology
