# Supplementary material for: The Effects of Ultra-High Pressure Combined with Egg White Protein on the Gel Physical Properties of Reduced-Salt Shrimp Surimi
Source: Foods. 2025 Jun 19;14(12):2144. doi: 10.3390/foods14122144 (PMC12191933; doi:10.3390/foods14122144)
Supplement: Supplementary file 1 [file foods-14-02144-s001.zip › foods-3688801-supplementary.pdf]

Table S1. The results of test and verify.

Groups	Gel strength (g·mm)	WHC (%)	Whiteness
1.5% NaCl	28.549±2.686 [b]	35.218±1.517 [c]	56.574±0.923 [c]
2.5% NaCl	48.735±3.388 [b]	38.239±1.189 [b]	60.673±0.370 [b]
UHP-EWP-1.5% NaCl	285.415±37.282 [a]	42.147±0.677 [a]	63.565±1.866 [a]
